# Facts about Honey

**Published:** 1888-09

**Authors:** 


### FACTS ABOUT HONEY.

Starch and sugar when eaten, undergo a digestive change before they are assimilated. In Honey this change has been made to a considerable extent by the bees. It is partly digested, easy of assimilation, and concentrated, and furnishes the same element of nutrition as sugar and starch —imparts warmth and energy.

As a medicine, honey has great value and many uses. It is excellent in most lung and throat affections, and is often used with great benefit in place of cod-liver oil. Occasionally there is a person with whom it does not agree, but most people can learn to use it with beneficial results. Children, who have more natural appetites, generally prefer it to butter. Honey is a laxative and sedative, and in diseases of the bladder and kidneys it is an excellent remedy. It has much the same effect as wine or sfimulants, without their injurious effects, and is unequalled in mead and harvest drinks. As an external application it is irritating when clear, and soothing when diluted. In many places it is much appreciated as a remedy for croup and colds. In preserving fruit, the formic acid it contains makes a better pceservative than sugar syrup, and it is also used in cooking and confectionery.—*Am. Bee Journal.*

